# Improving the Properties of Composite Titanium Nitride Layers on the AZ91D Magnesium Alloy Using Hydrothermal Treatment

**DOI:** 10.3390/ma14195903

**Published:** 2021-10-08

**Authors:** Michał Tacikowski, Janusz Kamiński, Krzysztof Rożniatowski, Marcin Pisarek, Rafał Jakieła, Paweł Marchlewski, Tadeusz Wierzchoń

**Affiliations:** 1 Faculty of Materials Science and Engineering, Warsaw University of Technology, ul. Wołoska 141, 02-507 Warsaw, Poland; janusz.kaminski@pw.edu.pl (J.K.); krzysztof.rozniatowski@pw.edu.pl (K.R.); tadeusz.wierzchon@pw.edu.pl (T.W.); 2 Institute of Physical Chemistry, Polish Academy of Sciences, ul. Kasprzaka 44/52, 01-224 Warsaw, Poland; mpisarek@ichf.edu.pl; 3 Institute of Physics, Polish Academy of Science, Al. Lotników 32/46, 02-668 Warsaw, Poland; jakiela@ifpan.edu.pl; 4 Sieć Badawcza Łukasiewicz Institute of Precision Mechanics, ul. Duchnicka 3, 01-796 Warsaw, Poland; pawel.marchlewski@imp.lukasiewicz.gov.pl

**Keywords:** magnesium alloys, titanium nitride, composite surface layers, hybrid method, hydrothermal treatment, overheated steam, corrosion and wear resistance

## Abstract

Coating magnesium alloys with nitride surface layers is a prospective way of improving their intrinsically poor surface properties; in particular, their tribological and corrosion resistance. These layers are usually produced using PVD methods using magnetron sputtering or arc evaporation. Even though the thus-produced layers significantly increase the wear resistance of the alloys, their effects on corrosion resistance are unsatisfactory because of the poor tightness, characteristic of PVD-produced products. Tightness acquires crucial significance when the substrate is a highly-active magnesium alloy, hence our idea to tighten the layers by subjecting them to a post-deposition chemical-hydrothermal-type treatment. This paper presents the results of our experiments with a new hybrid surface engineering method, using a final tightening pressure hydrothermal gas treatment in overheated steam of the composite titanium nitride layers PVD, produced on AZ91D magnesium alloy. The proposed method resulted in an outstanding improvement of the performance properties, in particular resistance to corrosion and wear, yielding values that exceed those exhibited by commercially anodized alloys and austenitic stainless 316L steel. The developed hybrid method produces new, high-performance corrosion and wear resistant, lightweight magnesium base materials, suitable for heavy duty applications.

## 1. Introduction

The aim of the present work was to improve the durability of protective nitride surface layers on magnesium alloys using a recently developed, novel surface engineering hybrid method, based on the concept of a final tightening of layers using pressure hydrothermal gas treatment [1], to produce corrosion and wear resistant composite lightweight magnesium base material. This becomes especially important in the context of the growing interest in a much wider use for light alloys in modern technology, especially the lightest of the metallic structural materials: magnesium alloys (~1.8 g/cm^3^). This creates the need for new, effective surface engineering solutions that would increase the application potential of such alloys. In addition to the high specific strength, the attractiveness of magnesium alloys lies in the unique combination of their low density and a range functional properties, such as effective screening of electromagnetic fields, good vibration damping, biocompatibility, and excellent castability, which allows mass production of complex details in a single die casting operation. The barrier for wide expansion of magnesium alloys is their poor performance properties, in particular low corrosion and wear resistance. Unlike aluminum or titanium alloys, no magnesium alloys that are resistant to corrosion in chloride environments are available. The increasing tendency to reduce the weight of products by making wider use of lightweight materials, driven by practical, economic, and environmental considerations (fuel and energy economy, combustion gases reduction) poses new challenges by putting new demands on magnesium alloys, which stimulate their dynamic development [2,3] to make them competitive with aluminum alloys, polymers, and steel. In consequence, the challenge for surface engineering of magnesium alloys is to search for original, effective solutions to overcome performance limitations and simultaneously achieve high corrosion and wear resistance [2,3]. Given the high chemical activity of magnesium, simultaneous mechanical and wear resistance is essential to ensure durability in heavy duty applications. The production of surface nitride layers on magnesium alloys seems to be a prospective path towards overcoming both their naturally poor corrosion and tribological surface properties, and achieving properties that would allow magnesium alloys to be used in precision tribological applications that require low surface roughness or involve exposure to severe corrosion conditions [4,5]. Alloys treated in this way could be used to produce moving parts for machines and devices [6], for instance lightweight sliding elements in advanced optical devices for aviation and aerospace industries. For more than two decades, the use of nitride surface layers in magnesium alloy surface engineering has attracted the attention of various authors [6,7,8,9,10,11,12,13,14,15,16,17,18,19]. Nitride layers on magnesium alloys are usually produced using PVD methods, (magnetron sputtering or arc evaporation) as they are the only alternative methods that allow precise control of layer thickness, roughness, and porosity. In earlier investigations, these methods were used to produce surface layers of various nitrides, in particular of metals such as aluminum, chromium, and titanium [6,7,8,9,10,11,12], while recent research has focused mainly on titanium nitride-based layers [13,14,15,16,17], which seem to be the most prospective, due to the simultaneous high corrosion and wear resistance, combined with a relatively low susceptibility to cracking. While the nitride layers considerably improve the wear resistance of magnesium alloys, [8,11,16] their effect on corrosion resistance has usually been unsatisfactory [6], given their insufficient tightness due to porosity and structural defects, typical for PVD methods [20], which makes application impractical [4]. The problem of the tightness of the surface nitride layer becomes critical when the substrate is a highly electrochemically active magnesium alloy and the surface layer is composed of relatively noble chromium or titanium nitrides, which are electricity conductors, leading to the risk of intensive galvanic corrosion [4,20,21]. In such a situation, even the smallest discontinuity in the layer, caused by the presence of porosity, structural defects, or mechanical damage (which in practice are statistically unavoidable), the corrosive environment penetrates through the layer to the substrate. In effect, galvanic corrosion cells are formed between the substrate and the nitride layer, resulting in particularly intensive pitting corrosion. This results in the cathode nature of the conductive nitride layers and the great difference in corrosion potentials between the highly active substrate, i.e., magnesium, and the relatively noble coating composed of nitrides, such as CrN or TiN [4,5]. There are a number of ways to prevent galvanic corrosion between nitride coatings and substrate that were investigated by various authors [20], who were mainly looking for ways to improve the tightness of the layers, but also to reduce the potential differential between the coating and the substrate. One should notice the relatively effective surface engineering concepts developed in recent years by Hoche et al. [4,13,14,15] for magnesium alloys, focusing on titanium nitrides, which combine improved tightness with the reduced corrosion potential achieved by alloying titanium nitrides with magnesium [4,13,14] or other elements [15]. Good tightness was initially achieved by designing composite layers with a multi-layer nitride-ceramics structure [4]. Tighter and more corrosion resistant layers of titanium nitride with increased magnesium or rare earth element contents were obtained by using new generation HiPIMS impulse magnetron sputtering technology [15]. Recently, significant progress in the corrosion resistance of AZ91D magnesium alloy was achieved by producing multi interface Al-TiAlN nanocomposite films [19]. Other promising solutions investigated in recent years in our laboratory were based on the concept of using a hybrid method to produce tighter, diffusive-type layers: first using chromium [12], which was then abandoned for titanium nitride surface layers [16], combined with a final tightening of the layers using the oxynitriding process under glow discharge conditions [16]. However, given that the related corrosion improvement, although relatively important in the case of titanium nitride, was associated with reduced tribological resistance of the layers [16], other solutions were sought that would ensure both high corrosion and wear resistance. Therefore, we proposed another method of chemical tightening of titanium nitride layers by oxides using hydrothermal treatment in a boiling water bath [21], which is a simple and easily available solution, commonly used in light alloy anodizing technology to seal newly produced light metal oxides layers, but never before used to tighten nitrides surface layers. Using this method, the composite titanium nitride layers on titanium and aluminum sub-layers on magnesium AZ91D alloy (TiN–Ti–Al), which was the multilayer structure specially designed to enable the tightening [21,22], were sealed with titanium oxides formed in a boiling water bath, yielding surprisingly good results, providing a radical improvement of corrosion resistance combined with a high wear resistance [5]. Further research, following the success of the method using hydrothermal tightening of nitride surface layers [21] that we developed, led us to propose a new concept based on the pressure gas variant of the hydrothermal sealing treatment of titanium nitride layers as a novelty solution covered by our related patent [1], the effects of which were investigated in the present work. The pressure hydrothermal gas sealing treatment consisted in processing the composite titanium nitride layer previously produced on magnesium alloy in overheated steam. The steam, gas phase of water medium used in this variant of the hydrothermal treatment, combined with the increased pressure of the sealing process was expected to stimulate the penetration of the medium into the layer and thus to improve the efficiency of the layers by tightening them with oxides in the deeper areas of the layers. It is worth to be noted that, in the recent works of Wu et al. [23,24], the hydrothermal type treatment, in combination with other surface treatment methods, was also successfully used in the biomedical area of magnesium alloys application to produce hydrothermal coatings, which ensure the combination of corrosion resistance and hydrophobic properties [23] or simultaneously improve corrosion resistance and cytocompatibility [24].

## 2. Materials and Methods

The composite hydrothermally sealed TiN-Ti-Al-type titanium nitride surface layers with a sub-layer of titanium and aluminum (TiN2Ti1Al10_SS) (Table 1) circa 10 µm thick, with nominal thicknesses of component layers at 2, 1, and 7 µm, respectively, were produced on die-casting AZ91D magnesium alloy containing 8.3–9.7 wt.% Al, 0.35–1.0 wt.% Zn, and 0.1 wt.% Mn, with Mg as the remaining balance. The main reason for choosing AZ91D magnesium alloy for this and all our previous works is its wide use in modern technology, accounting for over 90% of all magnesium alloy applications.

The design concept for the three-layer composite structure that would respond well to hydrothermal sealing had been developed and tested in earlier projects [21,22]. Choosing the thickness of the outside layer of titanium nitride was based on the results of earlier research [16]. It was assumed that the thickness values of the intermediate Ti and Al layers, which have the task of achieving property gradient between the hard external nitride layer and the relatively soft substrate, good adhesion of the titanium nitride (sub-layer Ti) and forming an anti-corrosion barrier (layer Al) responsive to hydrothermal treatment, should be 1 and 7 µm, respectively, relatively much higher for the Al barrier sub-layer, in order to prevent defects from penetrating across the sub-layer to the substrate.

**Figure 1:**
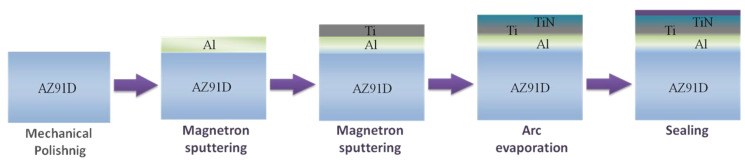
A schematic presentation of the experimental procedure of the hybrid process of producing composite titanium nitride TiN2Ti1Al10_SS layers with an intermediate titanium and aluminum sub-layer.

The layers were obtained using a four-stage hybrid method [5,21]. A schematic presentation of the experimental procedure, including uncoated and coated sample processing, is shown in Figure 1.

The method combines PVD-type deposition of an intermediate aluminum coating and a transition titanium coating, both by magnetron sputtering, followed by an outside titanium nitride coating produced by arc evaporation. Considering the high level of chemical activity and the relatively low melting point of magnesium, using PVD techniques to produce a specially designed, relatively complex composite structure of the TiN-Ti-Al titanium nitride layer was the only viable alternative technique. Unlike thermal spraying, PVD provides precise thickness control of the thin sub-layers, as well as roughness and porosity control. The layers were produced on mechanically polished rectangular plates (dimensions in [mm]: 2 × 10 × 3). Before the deposition of each subsequent coating, oxide passivation film was removed from the surfaces via ion etching in cathode sputtering process in the processing chamber. The intermediate layers, counting from the substrate: aluminum and titanium produced in laboratory magnetron sputtering device, working in DC mode, using aluminum and titanium targets of 99.5 and 99.6 purity, respectively (GfE GmbH), and the external layer of TiN-type titanium nitride layer in the arc evaporation MZ383 device (Metaplas Ionon, Bergisch Gladbach, Germany) manufacturerin a reactive process using titanium targets of 99.6 (GfE GmbH) and nitrogen atmosphere with 6N purity. The detailed parameters used in the each of the PVD process stages, developed in previous works [21], are given in Table 2.

The final stage of the hybrid method consisted of sealing the layers by pressure hydrothermal gas treatment performed in overheated steam [1]. The treatment of the AZ91 alloy samples covered with TiN-Ti-Al composite titanium nitride layers was performed in an autoclave that was used to produce overheated steam as a sealing medium. The sealing process, lasting between 10 to 60 min, was performed in the temperature range 110–150 °C. During the process, deionized water was used to produce steam. The pressure of the process was between 1.2 and 2.0 bars.

In order to assess how well the examined pressure hydrothermal gas treatment variant of the composite titanium nitride layers improved the performance properties of the AZ91D alloy (TiN2Ti1Al10_SS), several other coating–substrate variants and other reference materials, listed with their denotations in Table 1, were used for comparison. The previously studied variants [21] included the as-deposited (TiN2Ti1Al10) composite TiN-Ti-Al titanium nitride layers variant and the originally developed hydrothermally treated variant of the layers (TiN2Ti1Al10_S) sealed in a boiling water bath. The other materials used as reference to compare the achieved corrosion and wear resistance improvement included the untreated (AZ91D) and the commercially anodized AZ91D alloy (ANOD), as well as AISI 316L stainless steel (316L).

The characterization of the composite TiN-Ti-Al type titanium nitride layers’ microstructure involved cross section optical microscopy examinations, scanning electron microscopy (SEM) observations of the layers surface morphology (Hitachi SU70 scanning microscope, Hitachi, Osaka, Japan) and secondary ion mass spectrometry (SIMS) chemical composition analyses (Cameca IMS6F spectrometer, Cameca, Gennevilliers, France). SIMS measurement was conducted using a cesium (Cs^+^) primary beam and secondary ions as measured, as MeCs^+^ clusters were analyzed. The method of measuring oxygen as OCs^+^ clusters has been described elsewhere [25]. The chemical and phase composition of the surface of the titanium nitride layer subjected to hydrothermal treatment was examined using X-ray photoelectron spectrometry (XPS) with a Microlab 350 apparatus (Thermo Electron–VG Scientific, East Grinstead, UK). The results of earlier extensive microstructure investigations of the composite hydrothermally sealed TiN-Ti-Al type titanium nitride surface layers with the use of scanning electron microscopy (Hitachi SU70 scanning microscope, Hitachi, Osaka, Japan) examination were reported in our previous works [22]. The effect of the hydrothermal process on the surface roughness was examined by laser optical profilometry using a Wyko NT9300 Optical Profiling System (Veeco Instruments Inc., Plainview, New York, NY, USA) in the vertical scanning interferometry (VSI) mode. 

The corrosion resistance of the composite titanium TiN-Ti-Al surface layers that were processed hydrothermally in overheated steam, as well as those of other reference materials (Table 1), were examined with the use of potentiodynamic and electrochemical impedance spectroscopy (EIS) methods in a 0.5 M NaCl solution at room temperature, using the AUTOLAB PGSTAT 100 potentiostat in the trielectrode system (tested electrode-reference electrode (sce—saturated calomel electrode) and auxiliary electrode (platinum). Impedance spectroscopy tests were conducted with an AC signal with the amplitude of 20 mV and a frequency of 1 mHz–100 kHz. EIS methods were recorded in the potentiostatic mode at open circuit potential (EOCP). The samples were polarized at a potential variation rate of 0.2 mV/s. The impedance spectra were analyzed using the Boukamp’s EQUIVCRT software (ver. 4.9.007, Eco Chemie B.V, Utrecht, The Netherland). The spectra were presented in the form of a Nyquist plot. In order to elucidate the mechanisms of the corrosion-resistance improvement obtained for the hydrothermally processed composite TiN-Ti-Al-type titanium nitride layers on a titanium sub-layer with an aluminum sub-layer on AZ91D alloy (TiN2Ti1Al10_S and TiN2Ti1Al10_SS variants), and in particular to determine the role of the aluminum sub-layer in the sealing process, complementary corrosion behavior investigations in deionized water with the use of titanium TiN-Ti nitride layer on titanium sub-layer reference variant (TiN2Ti1)—deposited on AZ91D without the aluminum sub-layer (Table 2)—were performed. A complex characterization of the mechanical performance properties of the composite TiN-Ti-Al-type titanium nitride surface layers based on the Daimler-Benz scratch test and wear test was performed in our previous work [5]. Wear resistance examinations of the composite TiN-Ti-Al titanium nitride layer (TiN2Ti1Al10_SS), which was hydrothermally pressure processed in overheated steam, and the reference materials (Table 1) investigated during the present work, were performed following the same test procedure as in previous works [5,22], in accordance with the Polish Standard PN-82/H-04332 [26] using the modified Amsler method of the disk on block. The wear system, lubricated with oil (SAE 30), was composed of a tested plate sample (25 × 10 × 3 mm^3^) and a heat-treated 1045 steel disk (35HRC, diameter φ 35 mm, 10 mm thick) as the counter body. The tests were performed within a load range up to 200 N with a rotational speed of 200 revolutions/min for 1 h.

## 3. Results and Discussion

### 3.1. Microstructure and Chemical Composition of the Layers

**Figure 2:**
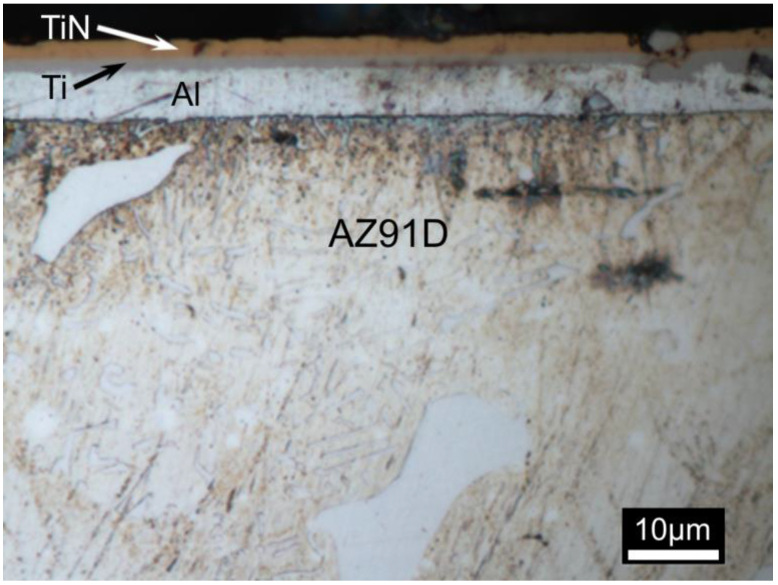
Microstructure of the composite TiN2Ti1Al10-type titanium nitride layer on magnesium AZ91D alloy.

The microstructure of the composite surface titanium nitride TiN–Ti–Al type layers with a sub-layer of titanium and aluminum (TiN2Ti1Al10), produced on AZ91D magnesium alloy using the hybrid method, combining magnetron sputtering of intermediate layers and cathode arc evaporation of the outside titanium nitride layer, is shown in Figure 2.

**Figure 3:**
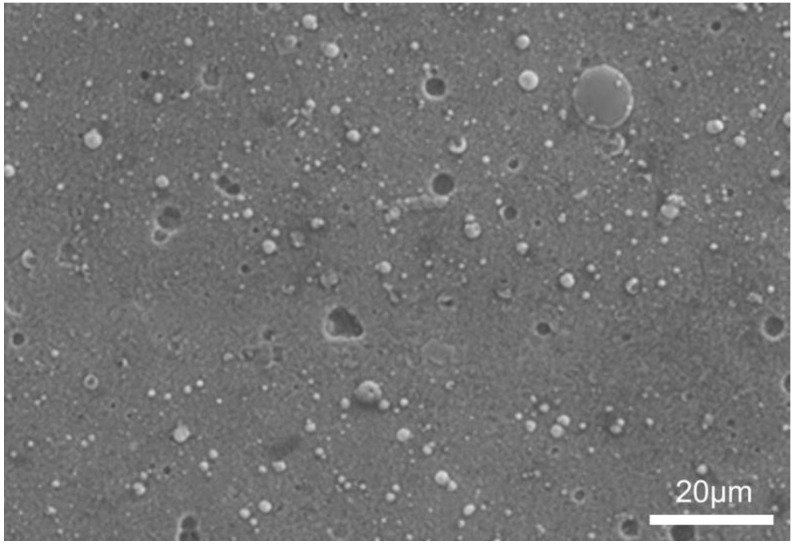
Surface morphology of the composite TiN2Ti1Al10 titanium nitride layer on magnesium AZ91D alloy—general view.

The thickness of the component layers starting from the surface was successively: surface titanium nitride layer (gold)—circa 2 µm; titanium sub-layer (grey)—circa 1 µm and aluminum (white)—circa 7 µm (Figure 2). Some defects in varying depth in the form of holes are visible in the surface titanium nitride layer. Neither of the sub-layers exhibited any visible discontinuities. These layers, which diffusively bound with the magnesium alloy, as shown by our earlier research [22], demonstrated good adhesion to the substrate and the absence of macroscopic defects or cross-layer discontinuities. The layers exhibited a surface morphology that is typical for the arc evaporation method used for the deposition of the outer titanium nitride, with characteristic defects, such as the so-called droplets and craters left after droplet decohesion (Figure 3), with craters being relatively shallow (Figure 2) and limited to the outside titanium nitride layer [21].

**Figure 4:**
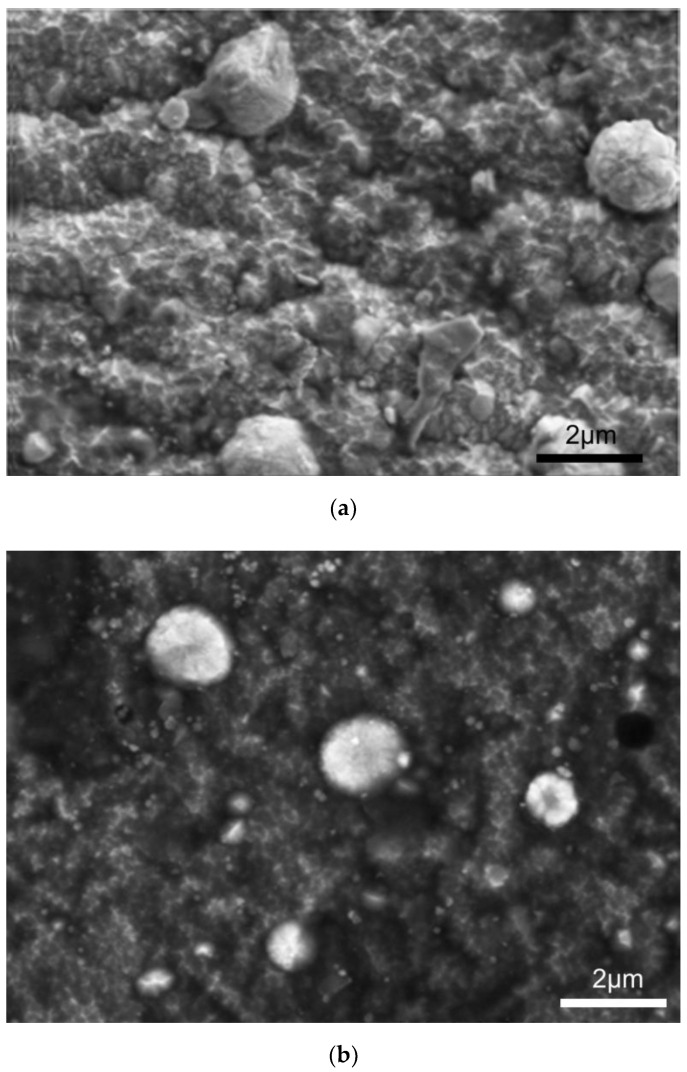
Surface morphology of the outside titanium nitride layer of the composite TiN2Ti1Al10 layer with modification produced by pressure hydrothermal treatment in overheated steam process: (**a**) before the process—as deposited (TiN2Ti1Al10) and (**b**) after the process (TiN2Ti1Al10_SS).

The hydrothermal treatment in overheated steam, as shown in Figure 4, modifies the surface morphology of the composite TiN-Ti-Al titanium nitride surface layers. The surface is covered with a thin film that is composed of a mixture of oxides and nitrides of titanium, as demonstrated by XPS analyses. The results of these analyses are summarized in Table 3.

Profilometry measurements indicated that the surface roughness changes from *R*_a_ (average arithmetic deviation of the roughness profile from the median line) 160 nm for the as-deposited layer variant (TiN2Ti1Al10), up to *R*_a_ 185 nm for the overheated steam variant (TiN2Ti1Al10_SS) and *R*_a_ 210 nm for the boiling water bath variant (TiN2Ti1Al10_S). This is due to the oxide film that formed during the hydrothermal treatment. However, although the oxide film produced in overheated steam slightly increased the surface roughness of the layers, its roughness was still lower (better for corrosion resistance) than that of the boiling water variant.

**Figure 5:**
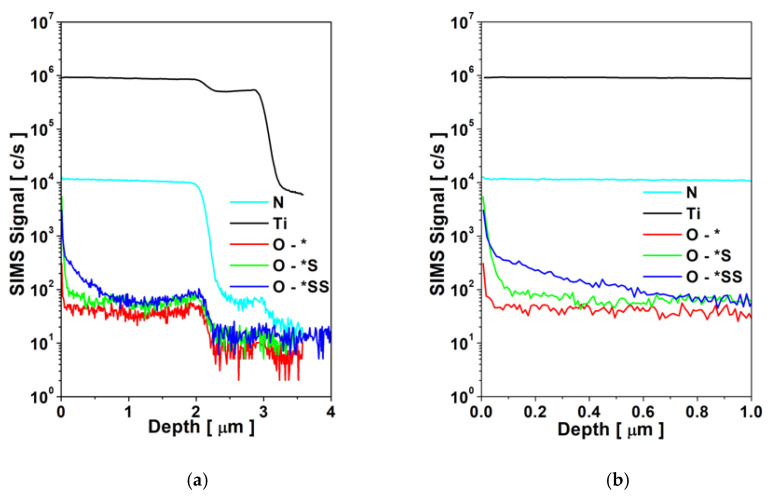
Comparison of oxygen distribution (SIMS) in the TiN-Ti zone of the composite TiN-Ti-Al type titanium nitride layers on AZ91D magnesium in as-deposited state and after hydrothermal treatment: O -*—as-deposited layer (TiN2Ti1Al10); O -*S—hydrothermally treated layer in boiling water bath (TiN2T11Al10_S); O -*SS—hydrothermally treated layer in overheated steam (TiN2T11Al10_SS): (**a**) distribution across the TiN-Ti near surface layer; (**b**) distribution in the vicinity of the surface.

The distribution of the elements in the TiN-Ti near-surface zone of the composite TiN2Ti1Al10-type titanium nitride layers, determined using secondary ion mass spectrometry (SIMS), is shown in Figure 5. The analysis demonstrates that the sealing hydrothermal treatment of the layers for both variants (TiN2Ti1Al10_S and TiN2Ti1Al10_SS) results in an increased oxygen concentration at the layer’s surface and in its vicinity. The oxygen penetration in the depth of the layer is clearly visible in the case of processing in the overheated steam (TiN2Ti1Al10_SS), while for processing in boiling water (TiN2Ti1Al10_S), the oxygen concentrates at the surface with only slightly pronounced penetration into the layer-surface vicinity. However, the increased concentration of oxygen persists for both sealing variants across the TiN titanium nitride surface layer and the titanium sub-layer, higher in the case of the TiN2Ti1Al10_SS variant.

### 3.2. Corrosion Behavior

**Figure 6:**
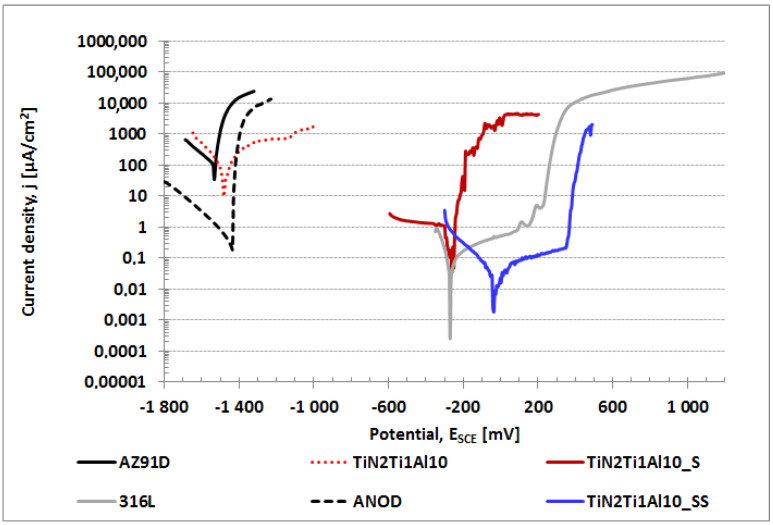
Polarization curves for uncoated (AZ91D) and anodized (ANOD) magnesium AZ91D alloy and for the alloy with various variants of composite TiN-Ti-Al titanium nitride layer: as-deposited (TiN2Ti1Al10) and sealed using hydrothermal treatment: in boiling water bath (TiN2Ti1Al10_S); in overheated steam (TiN2Ti1Al10_SS). For comparison, 316L stainless steel.

**Figure 7:**
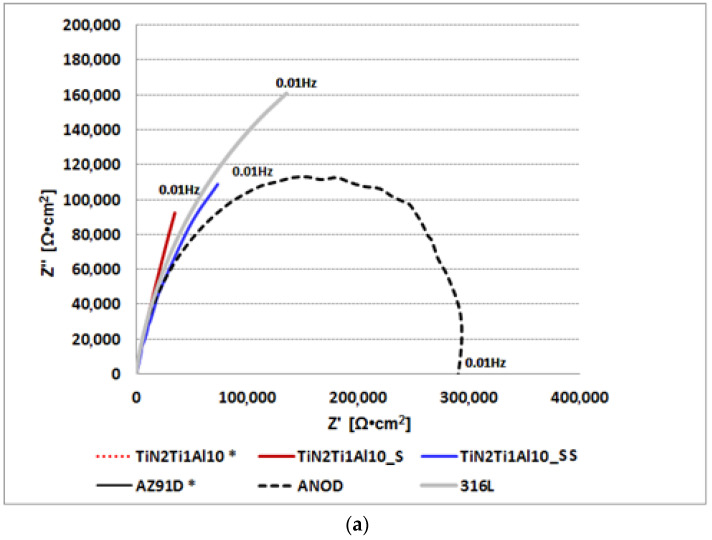

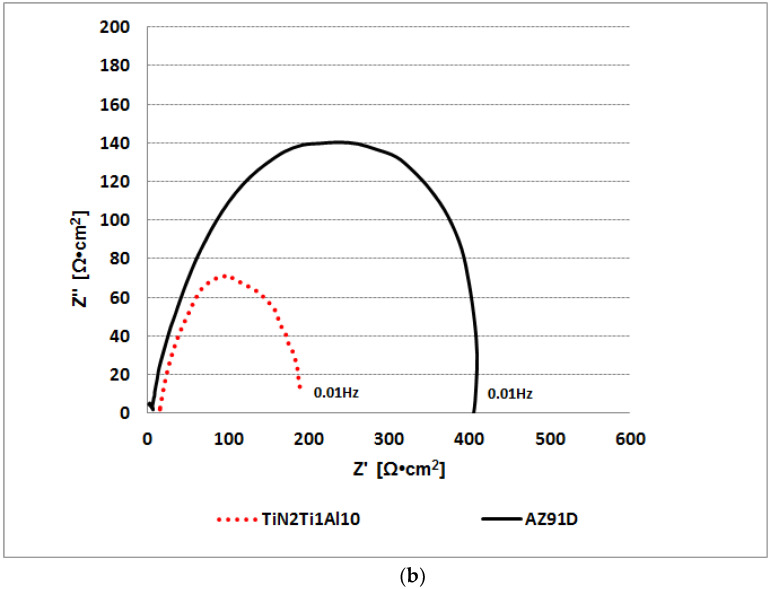
Impedance spectroscopy results obtained for the AZ91D alloy covered with various composite TiN-Ti-Al titanium nitride surface layers in the as-deposited (TiN2Ti1Al10) and sealed (TiN2Ti1Al10_S TiN2Ti1Al10_SS) variants and, for comparison, the untreated (AZ91D), anodized AZ91D alloy (ANOD) and 316L stainless steel (316L) Nyquist plots: (**a**) general view; (**b**) magnification for low impedance variants curves, not visible in Figure 7a: TiN2Ti1Al10* and AZ91D*.

The results of the corrosion potentiodynamic and electrochemical impedance spectroscopy investigation are shown, respectively, in Figure 6 and Figure 7. The corresponding corrosion parameters, such as corrosion potential and current (*E*_cor,_ *i*_cor_) values, as well as the breakdown potential (*E*_np_) obtained by potentiodynamic examination, are shown in Table 4. As seen in Figure 6, the formation of a composite TiN-Ti-Al titanium nitride surface layer on titanium sub-layer with a sub-layer of aluminum produced on AZ91D alloy in PVD process (TiN2Ti1Al10) did not result in the expected improvement of corrosion properties (Figure 6), even though the aluminum layer formed on AZ91D alloy by magnetron sputtering itself, as demonstrated in our previous works [23], showed relatively good corrosion resistance in 0.5 M NaCl (*E*_cor_ = −900 mV), and so was expected to improve the corrosion resistance as a sub-layer in the composite TiN-Ti-Al titanium nitride layer structure, acting as an anti-corrosive barrier able to stop the access of the aggressive environment to magnesium alloy. It was only after the final hydrothermal treatment in boiling water bath was performed [21] (TiN2Ti1Al10_S) that a radical improvement of corrosion resistance was observed, manifested by an unusually large shift of the corrosion potential towards positive values, i.e., by circa Δ*E*_cor_ = 1300 mV (Figure 6), with very high values of layer impedance [5]. Given the fact that the corrosion characteristics achieved for AZ91D alloy with the hydrothermally sealed composite TiN-Ti-Al titanium nitride layer was still not fully satisfying due to the absence of a passive range, research was undertaken to develop a new variant of pressure hydrothermal treatment performed in overheated steam (TiN2Ti1Al10_SS). The results of the treatment are shown in Figure 6.

Hydrothermal treatment in overheated steam results in an unusually large (for magnesium alloys) shift in the corrosion potential to the value of circa *E*_cor_ = −40 mV, i.e., by almost Δ*E*_cor_ = 1500 mV, relative to unprotected AZ91D alloy with a titanium nitride layer (AZ91D) with *E*_cor_ = −1530 mV. The resulting increase of corrosion potential is circa Δ*E*_cor_ = 220 mV higher than in the case of hydrothermal treatment in boiling water bath and is associated with the corrosion current decrease by an order of magnitude at circa *i*_cor_ = 0.022 µA/cm^2^. This demonstrates a more efficient tightening of the layer in the overheated steam process. The main benefits from processing in overheated steam is the resulting relatively broad (ca. 300 mV large) passive range with a positive breakdown potential of circa *E*_np_ = 350 mV, resulting in corrosion resistance that is much better than that of the commercially anodized AZ91D alloy (ANOD) (Figure 6), which despite of the relatively low value of corrosion potential *E*_cor_ = −1445 mV does not exhibit any passive behavior. Moreover, what may be considered as the most significant achievement of the new solution, the corrosion resistance of the composite TiN-Ti-Al titanium nitride surface layers on the AZ91D alloy treated in overheated steam is better than that of 316L stainless steel (Figure 6). The polarization test results are consistent with the results of impedance spectroscopy tests (Figure 7), showing high impedance values for Z′, Z″, comparable to those of stainless steel, and higher than those of commercially anodized alloy; thus, confirming the achieved high corrosion resistance.

The outstanding improvement of corrosion behavior of the composite TiN-Ti-Al titanium nitride layers (which, typically for PVD products, are highly sensitive to galvanic corrosion) achieved by using hydrothermal treatments in overheated steam (TiN2Ti1Al10_SS) described in the preceding section (Figure 6 and Figure 7) and may be attributed to the effective tightening of the layer with the products of the chemical reaction induced by the hydrothermal treatment. SIMS analysis (Figure 5) reveals that the oxygen concentration at the surface was about two orders of magnitude higher than in the core of the layer. In fact, during this treatment, as indicated by the XPS analysis, the surface of the layer is covered with titanium oxides (Table 3) that form a thin sealing film (Figure 4).

As shown by laser optical profilometry tests, the oxide film modifies the layer’s surface roughness, with *R*_a_ increased by circa 30% for the hydrothermal treatment in boiling water bath (TiN2Ti1Al10_S), compared to only circa 15% for the treatment in overheated steam (TiN2Ti1Al10_SS). The oxide layer thickness seems thus to be more uniform in the latter case. The lower surface development is also more favorable in terms of corrosion resistance. However, the most efficient improvement of corrosion resistance of the composite titanium nitride layer on AZ91D was observed in the case of hydrothermal processing in overheated steam and is certainly primarily related to the more effective tightening of the layer with titanium oxides produced in reaction with the overheated steam; not only on its surface, but most likely also inside the discontinuities related to droplets and craters penetrating into the depths of the layer. This is clearly confirmed by SIMS analysis (Figure 5) which shows that the processing in overheated steam (TiN2Ti1Al10_SS) results in higher oxygen concentration across the layer, compared to both the as deposited variant (TiN2Ti1Al10) and the boiling water processing variant (TiN2Ti1Al10_S). Moreover, in the TiN2Ti1Al10_SS layer in the near surface zone of circa 0.5 µm the concentration of oxygen is significantly increased, compared to the deeper zones, whereas in the TiN2Ti1Al10_S the higher oxygen concentration is limited to only the surface.

In fact, the increased pressure during hydrothermal treatment in overheated steam (TiN2Ti1Al10_SS) facilitates steam penetration deep into the composite titanium nitride layer along the discontinuities and resulting in oxidation; hence, titanium oxide also forms inside the layer and seals the discontinuities. The appearance of a passive range on the polarization curve in positive corrosive potential areas produced by treatment in overheated steam (Figure 6) is most probably the result of sealing with oxides inside the layer at the level of the titanium sub-layer. It is worth noting that, in the case of titanium, the advantage is the relatively high Pelling–Bedworth ratio, close in value to 1.8, which should favor an efficient, tight filling of the discontinuities inside the layer with titanium oxides. Moreover, titanium is commonly known to have passivation capabilities in chloride environments and thus demonstrates high anticorrosion properties. In consequence, it is just the titanium sub-layer sealed in hydrothermal process treatment that most probably serves as the effective anticorrosion barrier that controls the corrosion behavior of the composite titanium nitride layer treated with the overheated steam.

In the case of the composite titanium nitride layer processed in the boiling water bath (TiN2Ti1Al10_S), as shown by SIMS examination (Figure 5), the tightening of the layer occurs predominantly on the surface, which explains the related lack of passive behavior (Figure 6). In fact, in this case, whenever the oxides film, which tightens the layer’s surface, breaks down due to corrosive processes or mechanical damage, the layer is no longer sealed inside and is automatically exposed directly to chloride environment penetration through the titanium and aluminum sub-layers defects towards the magnesium alloy substrate, so it behaves like an as-deposited layer (Figure 6), which is sensitive to intensive pitting corrosion. Even though the aluminum layer on magnesium alloy on its own is relatively corrosion resistant [22], as a sub-layer of the composite TiN-Ti-Al titanium nitride layer, neighboring directly with the titanium sub-layer as shown previously [27], it does not constitute a durable anti-corrosion barrier that would be able to effectively isolate the outer, relatively noble, titanium nitride coating from the highly active magnesium base substrate. This is most probably due to the formation of corrosion cells between the aluminum sub-layer and the titanium sub-layer, resulting in fast galvanic corrosion [27]. In consequence, it leads to the perforation of the aluminum sub-layer, exposing the magnesium AZ91D alloy substrate to the corrosive environment, and the formation of aluminum–magnesium cells, which accelerate galvanic corrosion processes, as magnesium is even more active than aluminum. In the case of the layer processed in overheated steam (TiN2Ti1Al10_SS), once the oxide film tightening the layers surface breaks down, the penetration of the chloride environment is stopped at the level of the sealed and corrosion resistant titanium sub-layer.

**Figure 8:**
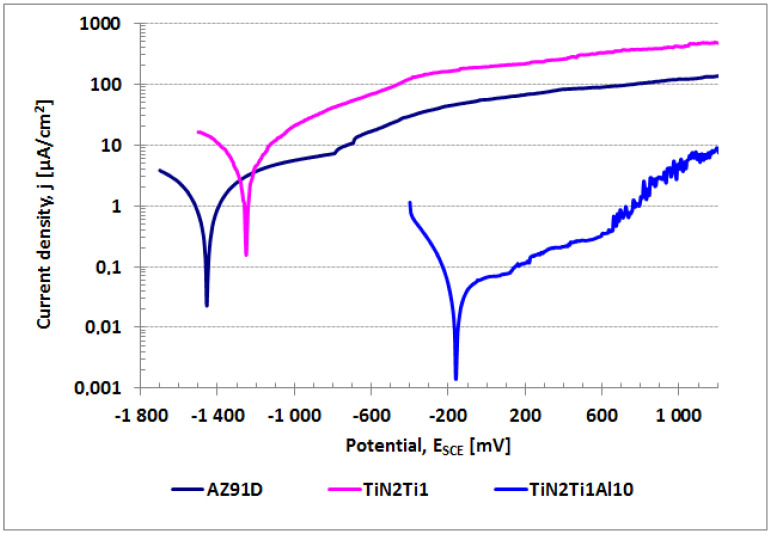
Polarization curves in deionized water for magnesium AZ91D alloy uncoated (AZ91) and coated with various types of titanium nitride surface layers: TiN-Ti-Al type with a titanium and aluminum sub-layer and, TiN-Ti type with titanium and without aluminum sub-layer, both in as-deposited variants (TiN2Ti1Al10 and TiN2Ti1_S, respectively).

This leads to the conclusion that the effective sealing deep inside of the outside titanium nitride layer, and in particular of the titanium sub-layer, such as was obtained in the overheated steam hydrothermal treatment variant, is essential to effectively isolate the aluminum sub-layer on magnesium alloy from the environment, and therefore to eliminate the risk of galvanic cells formation to prevent the accelerated galvanic corrosion of the AZ91D alloy covered with the composite titanium nitride layer. Even though, contrary to the original assumptions [21], the aluminum sub-layer as shown earlier [27] does not act as an anti-corrosion barrier in hydrothermally processed composite TiN-Ti-Al titanium nitride layer, its presence in the structure of the layer is a key factor in making the tightening process effective. It is supposed [22] that role is to prevent any galvanic corrosion processes between the titanium nitride layer and the AZ91D magnesium alloy substrate during hydrothermal treatment in the boiling water bath, which could precede and in consequence effectively prevent the successful tightening of the composite layer with oxides. The results of the complementary corrosion tests in deionized water (normally used for hydrothermal treatment) performed in this work (Figure 8) definitely confirm the latter hypothesis. In fact, when the titanium nitride layer on the titanium sub-layer was directly produced on AZ91D magnesium alloy (TiN2Ti1) it was almost as active as the AZ91D alloy and thus highly susceptible to corrosion in deionized water, and not only in the as-deposited variant (Figure 8). The increased temperature of 100 °C during the hydrothermal treatment undoubtedly facilitates corrosion processes in deionized water, which for magnesium normally occurs at pH < 1 and macroscopically leads to the formation of relatively numerous pits on the layer’s surface. However, while the outer titanium nitride layer on the titanium sub-layer is separated from magnesium alloy by an aluminum sub-layer (TiN2Ti1Al10), the corrosion in deionized water is inhibited and thus the sealing via hydrothermal treatment may be effective (Figure 8).

### 3.3. Mechanical Properties

**Figure 9:**
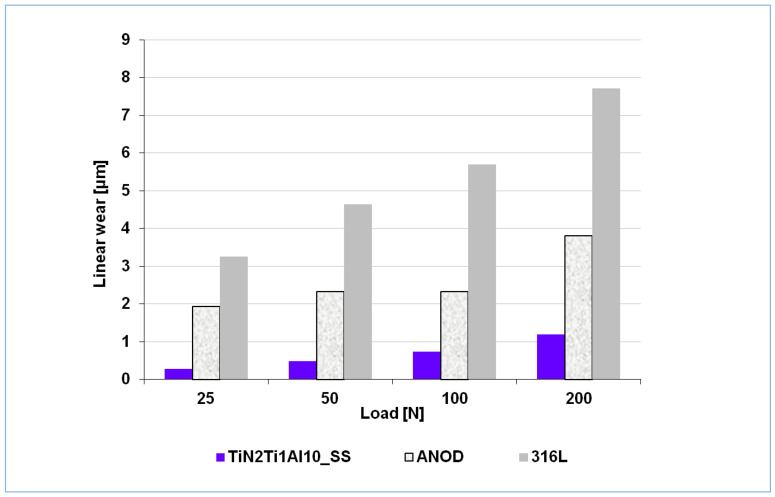
Wear resistance in Amsler test of the composite titanium nitride layer (TiN2Ti1Al10_SS) compared to stainless 316L steel and commercially anodized AZ91D magnesium alloy.

A complex characterization of the mechanical performance properties of the composite TiN-Ti-Al type titanium nitride layers was performed during our previous work [5] and demonstrated that the layers, not only significantly improved corrosion resistance, but also mechanical damage resistance to factors such as exposure to concentrated point loads, scratching, or wear. None of these exposures resulted in cracks or layer decohesion. In fact, the layer’s resistance to mechanical continuity failure is a key advantage of the developed composite titanium nitride layers given the critical requirement of absolute tightness of the layers, as noted previously [21,22], related with their cathodic nature and the highly active magnesium substrate, thus highly susceptible to galvanic corrosion. However, it is worth noting that, for the reasons discussed above, in the case of the composite TiN-Ti-Al type titanium nitride layers subjected to the hydrothermal treatment in the overheated steam (TiN2Ti1Al10_SS), which are sealed deep inside the titanium sub-layer, even if the continuity of either the tightening film on the layer’s surface or the outer nitride zone fails mechanically, it would not be critical from the corrosion resistance point of view. The mechanical resistance of the composite TiN-Ti-Al type titanium nitride layers in wear condition is much higher than commercially anodized AZ91D alloy; in fact, it is comparable to the resistance of typical bearing 100Cr6 steel [5]. Moreover, the wear resistance examinations of the composite TiN-Ti-Al titanium nitride layer hydrothermally pressure processed in overheated steam (TiN2Ti1Al10_SS) and the reference materials (Table 1) investigated during the present work (Figure 9) showed that the coated AZ91D magnesium alloy exhibits wear resistance much higher than the reference stainless 316L steel.

High mechanical resistance of the TiN-Ti-Al type layers guarantees high functional durability in those service conditions where composite titanium nitride layers on AZ91D magnesium alloy are exposed to both corrosive and mechanical factors. This is of crucial importance, taking the risk of galvanic corrosion in case of mechanical failure of the layers into account. What needs to be emphasized is the superiority of the variant of hydrothermal processing of the composite titanium nitride layers in overheated steam (TiN2Ti1Al10_SS) over treatment in a boiling water bath (TiN2Ti1Al10_S) in terms of mechanical durability of the layers. This is related to the fact that in this case the corrosion resistance, as discussed above, is controlled by the tightness of the titanium sub-layer, which is less sensitive to any damage that could occur in the titanium oxide film tightening the surface or, even more, in the titanium nitride outer zone.

## 4. Conclusions

1. Corrosion resistance of the composite TiN-Ti-Al titanium nitride layers with titanium and aluminum sub-layers produced on AZ91D magnesium alloy using the PVD hybrid method, may be radically improved by applying a final tightening procedure using a pressure hydrothermal gas treatment performed in overheated steam (recently patented), which results in a better corrosion resistance than that of 316L stainless steel.
2. The outstanding improvement of the composite TiN-Ti-Al titanium nitride layers corrosion resistance processed in overheated steam may be attributed to the effective deep sealing inside the layers, and in particular, on the level of the titanium sub-layer, which actually became a corrosion barrier controlling the behavior of the entire layer. Moreover, as a direct consequence, the as-sealed composite layer has the crucial advantage of reducing the risk of galvanic corrosion once any mechanical damage in service conditions occurs in the titanium oxide film tightening the surface of the layer or in the titanium nitride outer zone, breaking their continuity. The susceptibility to galvanic corrosion increases when the corrosion barrier is limited to a surface titanium oxide film, like the one produced using the originally developed hydrothermal treatment variant in a boiling water bath.
3. The corrosion resistance of AZ91D alloy covered by the composite TiN-Ti-Al titanium nitride layers hydrothermally sealed in overheated steam is significantly higher than that of the commercially anodized AZ91D alloy, and also much higher than when composite TiN-Ti-Al titanium nitride layers are tightened by hydrothermal treatment in a boiling water bath. The key advantage of the composite titanium nitride layers on the AZ91D alloy that is hydrothermally sealed in an overheated steam is their passivity in the relatively large range with the positive breakdown potential, obtained thanks to the anticorrosion properties of the effectively sealed titanium sub-layer.
4. In addition to its high corrosion resistance, the composite titanium nitride layers on the AZ91D alloy exhibit high resistance to wear which significantly exceeds the wear resistance of 316L stainless steel and anodized AZ91D alloy. The newly developed hybrid method that involves a key operation of final sealing of the nitride layers in overheated steam therefore allows to produce high performance, both corrosion and wear resistant, lightweight magnesium base material suitable for wider applications.

## Figures and Tables

**Table 1:** Investigated variants of layers on AZ91D alloy and reference materials with denotation.

Denotation	Layers Variant	Sub-Layer Layer Type and Thickness [µm]			Treatment	Sealing Process Medium
		TiN	Ti	Al		
TiN2Ti1Al10_SS	TiN-Ti-Al	2	1	7	Hydrothermal sealing	Supersaturated steam
TiN2Ti1Al10_S	TiN-Ti-Al	2	1	7	Hydrothermal sealing	Boiling water bath
TiN2Ti1Al10	TiN-Ti-Al	2	1	7	As deposited	-
TiN2Ti1	TiN-Ti	2	1	–	As deposited	-
AZ91D *	AZ91D alloy, uncoated	-			As delivered	-
ANOD *	AZ91D anodized alloy	-			Commercial anodizing	Boiling water bath
316L *	316L steel, uncoated	-			As delivered	-

* Reference material.

**Table 2:** PVD processes parameters used to produce the investigated layers.

Parameter	Ion Etching		Deposition		
			Al	Ti	TiN
Source	Arc, titanium cathode	Magnetron, argon	Magnetron	Magnetron	Arc, titanium cathode
Current	50 A	5 A	5 A	5 A	50 A
Bias	600 V	800 V	100 V	100 V	100 V/10 kHz
Substrate temperature	circa 200 °C	<200 °C	<200 °C	<200 °C	circa 200 °C
Pressure	1.2 × 10^−2^ mbar	5 × 10^−3^ mbar	5 × 10^−3^ mbar	5 × 10^−3^ mbar	1.2 × 10^−2^ mbar

**Table 3:** Binding energy peaks and the corresponding phase composition of the composite titanium nitride layers’ surface subjected to hydrothermal treatment in overheated steam (XPS). Normalized results.

Peak	Binding Energy [eV]	At.%	Phase
Ti2p3	455.2	40.0	TiN_2_
Ti2p3	458.6	33.9	TiO_2_
Ti2p3	456.9	26.1	Ti_2_O_3_
		Σ 100.0	

**Table 4:** Values of corrosion parameters in potentiodynamic tests (Figure 6): corrosion potential, *E*_cor_; current density—*I*_cor_; breakdown potential—*E*_np_; current density—*I*_np_.

Variant Tested	Parameter			
	*E*_cor_ [mV]	*i*_cor_ [μA/cm^2^]	*E*_np_ [mV]	*i*_np_ [μA/cm^2^]
TiN2Ti1Al10_SS	−27	0.033	+350	0.028
TiN2Ti1Al10_S	−260	0.56	−	−
TiN2Ti1Al10	−1480	50	−	−
AZ91D	−1530	102	−	−
ANOD	−1450	0.27	−	−
316L	−270	0.085	+220	0.095

## Data Availability

All data included in this study are available upon request by contact with the corresponding author.
